# Effects of bilateral lung transplantation on cardiac autonomic modulation and cardiorespiratory coupling: a prospective study

**DOI:** 10.1186/s12931-021-01752-6

**Published:** 2021-05-21

**Authors:** E. Tobaldini, G. D. Rodrigues, G. Mantoan, A. Monti, G. Coti Zelati, Ludovico Furlan, P. Tarsia, L. C. Morlacchi, V. Rossetti, I. Righi, L. Rosso, M. Nosotti, P. P. S. Soares, N. Montano, S. Aliberti, F. Blasi

**Affiliations:** 1 Department of Internal Medicine, Fondazione IRCCS Ca’ Granda Ospedale Maggiore Policlinico, 20122 Milan, Italy; 2 Department of Clinical Sciences and Community Health, University of Milan, Francesco Sforza St, 35, 20122 Milan, Italy; 3 Department of Physiology and Pharmacology, Biomedical Institute, Fluminense Federal University, Niterói, 24210-130 Brazil; 4 Respiratory Unit and Cystic Fibrosis Adult Center, Fondazione IRCCS Ca’ Granda Ospedale Maggiore Policlinico, 20122 Milan, Italy; 5 Department of Pathophysiology and Transplantation, University of Milan, 20122 Milan, Italy; 6 Thoracic Surgery and Lung Transplantation Unit, Fondazione IRCCS Ca’ Granda Ospedale Maggiore Policlinico, 20122 Milan, Italy

**Keywords:** Heart rate variability, Cardiac autonomic modulation, Lung transplantation, Spectral analysis, Symbolic analysis

## Abstract

**Background:**

Although cardiac autonomic modulation has been studied in several respiratory diseases, the evidence is limited on lung transplantation, particularly on its acute and chronic effects. Thus, we aimed to evaluate cardiac autonomic modulation before and after bilateral lung transplantation (BLT) through a prospective study on patients enrolled while awaiting transplant.

**Methods:**

Twenty-two patients on the waiting list for lung transplantation (11 women, age 33 [24–51] years) were enrolled in a prospective study at Ospedale Maggiore Policlinico Hospital in Milan, Italy. To evaluate cardiac autonomic modulation, ten minutes ECG and respiration were recorded at different time points before (T0) and 15 days (T1) and 6 months (T2) after bilateral lung transplantation. As to the analysis of cardiac autonomic modulation, heart rate variability (HRV) was assessed using spectral and symbolic analysis. Entropy-derived measures were used to evaluate complexity of cardiac autonomic modulation. Comparisons of autonomic indices at different time points were performed.

**Results:**

BLT reduced HRV total power, HRV complexity and vagal modulation, while it increased sympathetic modulation in the acute phase (T1) compared to baseline (T0). The HRV alterations remained stable after 6 months (T2).

**Conclusion:**

BLT reduced global variability and complexity of cardiac autonomic modulation in acute phases, and these alterations remain stable after 6 months from surgery. After BLT, a sympathetic predominance and a vagal withdrawal could be a characteristic autonomic pattern in this population.

**Supplementary Information:**

The online version contains supplementary material available at 10.1186/s12931-021-01752-6.

## Background

Pulmonary transplantation is the life-saving standard of care for patients affected by end-stage lung disease. A growing number of transplantations is performed every year and great progress has been made in terms of surgical procedure, immunosuppression and medical innovations. Despite this, mortality in lung recipients is still significant: infections, graft dysfunction, and chronic rejection have a major role in the first year after transplant, but also cardiovascular events are a relevant cause of death [1].

Respiratory and cardiovascular systems share a strong reciprocal relationship, partly mediated by the autonomic nervous system [2]. Respiratory pacemaker cells, located in the brainstem, generates a rhythm whose breathing frequency and depth are mainly controlled by central and peripheral chemoreflexes influencing the vagal modulation to the heart. Also, breathing and lung amplitude stimulate stretch lung receptors producing an inhibitory signal to the vagal nerve in the brainstem. Cardiac pacemaker cells are also modulated by central oscillators, which interacts with rhythms caused by baroreflex and breathing oscillations [3]. Indeed, it was widely documented in current literature that breathing is a powerful modulator of heart rate variability (HRV) [4]. HRV analysis has proven to be a reliable, non-invasive method to investigate the autonomic neural modulation of cardiovascular functions, and a decline of HRV has shown to be related to higher cardiovascular risk and poor prognosis [5, 6].

Current evidence of the effects of pulmonary transplantation on cardiovascular regulation are very limited [7–14]. It is known that lung transplant recipients show an increased heart rate at rest. It is also known that the surgical procedure for bilateral lung transplantation (BLT) may per se determine damage to lung afferent cardiovascular innervation [7]; this could result in an interruption of autonomic pathways and a consequent loss of respiratory modulation of heart rate. Moreover, few studies were conducted to assess cardiac autonomic modulation in lung transplant recipients [7–12]. For instance, Berakis et al. [13] and Fontolliet et al. [14] compared lung transplant patients and healthy controls and suggested the existence of an altered autonomic balance, with a stronger sympathetic modulation. However, the major limitation of these studies is that transplant patients were compared to healthy controls.

The study aimed to investigate the acute and chronic effects of BLT on cardiac autonomic modulation through different tools of HRV analysis, testing the hypothesis that BLT reduces the global autonomic modulation to the heart, shifting the cardiac autonomic balance to a sympathetic predominance and a vagal withdraw.

## Methods

### Sample

This was a prospective, observational study on adult patients who were followed up in the Lung Transplant Program of the Ospedale Maggiore Policlinico Hospital (Milan, Italy) and underwent a BLT from January 2016 and January 2019.

The study protocol was approved by the Ethics Committee of Fondazione IRCCS Ca’ Granda Ospedale Maggiore Policlinico (Milan, Italy; protocol number 181, January 2017, 749–2016 bis) and was developed in compliance with the Declaration of Helsinki. Patients were enrolled. An informed written consent was signed by every patient before participation in this research.

Inclusion criteria were: (1) adults (> = 18 years); (2) patients in the lung transplant waiting list; (3) stable clinical condition (at least 4 weeks far from either exacerbations or antibiotics or hospitalization). The exclusion criteria for patients were at least one among the following: (1) absence of stable sinus rhythm on ECG, (2) number of extra systoles greater than 5% on ECG, (3) pacemaker rhythm, (4) non-invasive mechanical ventilation. We enrolled twenty-two patients at baseline and they completed the protocol of two recordings, baseline (T0) and acute phase (fifteen days; T1). Out of these 22 patients, 8 were lost at 6 months follow up (T2) due to ongoing hospitalization and/or acute events at the timing of recording. Thus, 14 patients completed the protocol at the 3 time points (T0–T1–T2).

### Experimental design

To evaluate cardiac autonomic modulation, we used a telemetric system device (LAB 3, Marazza Elettronica, Monza, ITA), which recorded simultaneously ECG (lead II) and respiratory thoracic movements through a thoracic piezoelectric belt to assess respiratory frequency (Hz). Each patient was recorded for 10 min in supine position (SUP) and 10 min in active orthostatic standing (ORT). Patients were not allowed to talk during the recording, and they were in spontaneous breathing.

For each patient, recordings were performed at different time points: before transplantation (T0), in occasion of a medical examination scheduled by the Lung Transplant Program, fifteen days (T1) and 6 months (T2) after transplantation.

The primary endpoint was to test the effects of BLT on sympathetic and vagal modulation on HRV in acute phase, i.e. 15 days after the surgery, in a population of adult patients; as a secondary endpoint, we tested the hypothesis that BLT impacts on chronic sympathetic and vagal modulation on HRV, 6 months after the transplant.

### Heart rate variability

To evaluate cardiovascular autonomic modulation, segments of 300 cardiac beats were processed through a specific software for R-R interval analysis (Heart Scope II, Amps LLC, New York, USA). For each registration, we analyzed both a segment at rest and a segment in orthostatic position. Autonomic dynamic response to orthostatic stress was also assessed through calculating the percentage change ∆ORT% [(HRV in SUP position − HRV in ORT position)/HRV in SUP position] for every parameter.

Spectral analysis identifies rhythmic oscillatory components of HRV. An autoregressive model was adopted, with a Hanning window and 50% overlap to obtain the spectral power in the low frequency component (LF, frequencies in the band 0.04–0.15 Hz) and high frequency component (HF, frequencies in the band 0.15–0.40 Hz). LF and HF were expressed in normalized units (LFnu and HFnu), which are obtained dividing each band power by the total power minus the very low frequency component (VLF, frequencies < 0.04 Hz). While LF is commonly considered a marker of both sympathetic and parasympathetic modulation, HF is a parasympathetic marker. LF/HF ratio was adopted to expresses the sympatho-vagal balance [15].

Also, a more recent nonlinear method, symbolic analysis, was adopted to evaluate HRV dynamics. Symbolic analysis divides consecutive R-R intervals in patterns of three symbols; each pattern is then classified into three families: 0 V, no variation (three equal symbols); 1V, one variation (two equal consecutive symbols and the remaining one different); 2V, two variations (three different symbols, with 2 like variations, 2LV, or 2 unlike variations, 2UV). While 0V is interpreted as a marker of sympathetic modulation, 2LV and 2UV are generally considered parasympathetic markers. The interpretation of 1V is still discussed. 0V, 1V, 2LV and 2UV are all expressed as percentages. Non-linear analysis is better able to detect non-reciprocal variations of sympathetic and parasympathetic modulations [16].

HF component of spectral analysis is usually synchronous with respiratory rate. To assess this relationship, we calculated the squared coherence function at high frequencies (HFk^2^) between breath rate and heart rate for each patient. RR-RESP HFk^2^ ranges from 0 (no correlation) and 1 (highest correlation) [17].

HRV regulation was also assessed through entropy-derived parameters, which measure cardiac complexity. Pathological situations and aging determine the dominance of one cardiovascular regulatory system on the others, thus reducing entropy and complexity of cardiac autonomic modulation. Corrected conditional entropy (CE) measures the quantity of information carried by R–R samples and consequent HRV predictability: it could range from 0 (future R-R values completely predictable) to 1 (future R–R values completely unpredictable). Index of regularity (Ro) is derived from corrected conditional entropy and could range from 1 (highest regularity and lowest complexity) to 0 (lowest regularity and highest complexity) [18].

### Statistical analysis

The normality of the samples was evaluated through the Shapiro–Wilk test, and non-parametric tests were used to analyse non-normally distributed data.

To evaluate the acute effects of lung transplantation, 22 patients were evaluated at T0 and T1. HRV comparison between the two-time points was performed by a paired t-test (p < 0.05, α = 0.05) or by Mann–Whitney U.

To evaluate the acute and chronic effects of lung transplantation, 14 patients were evaluated at different time points (T0, T1, T2). One-Way Analysis of Variance (p < 0.05, α = 0.05) was performed to compare each group of parameters at selected time points; for non-normally distributed data, Kruskal–Wallis One Way Analysis of Variance on Ranks was performed. A post-hoc analysis for all the comparison with a p value < 0.05 was performed (Holm-Sidak method). Data are reported as median and interquartile range (IQR 25–75%). For this study SigmaPlot, 12.0 (Systat Software Inc., San Jose, USA) was used. The software used to calculate sample power was G-Power version 3.1.9.2 (Heinrich-Heine-Universität Düsseldorf, Düsseldorf, Germany).

## Results

### Study population

Twenty-two patients [11 women; median age: 33 years (24–51)] on the waiting list for transplant were enrolled in this study. Fourteen patients were affected by cystic fibrosis, two by idiopathic pulmonary fibrosis, two by chronic obstructive pulmonary disease, and four were diagnosed with other indications for pulmonary transplant. Demographic characteristics, cardiovascular risk factors, respiratory function, chronic lung infections, and medications of patients enrolled in this study are listed in Table 1. Among enrolled patients, every patient was registered at T0 and T1. Fourteen patients were registered also at T2, 6 months after transplant.

**Table 1:** Demographics, cardiovascular risk factors, respiratory function, chronic lung infections and medications of patients enrolled in this study

	Study population n = 22
Demographics, n (%)	
Age, median (IQR) years	33 (24–51)
Female	11 (50)
Body mass index, median (IQR)	20.8 (18.2–23.9)
Lung Allocation Score, median (IQR)	34.6 (32.8–38.6)
Cystic fibrosis	14 (63.6)
Idiopathic pulmonary fibrosis	2 (9.1)
Chronic obstructive pulmonary disease	2 (9.1)
Nonspecific interstitial pneumonia	1 (4.5)
Other indications for lung transplant	3 (13.6)
Cardiovascular risk factors, n (%)	
Hypertension	4 (18.2)
Diabetes	10 (45.5)
Pulmonary hypertension	5 (22.7)
mPAP, median (IQR) mmHg	21.5 (18–24)
Respiratory function, median (IQR)	
Exacerbations, n (%)	16 (72.7)
P/F	319 (279–342)
pCO2, mmHg	41 (38.8–48)
FEV1% of predicted	28.5 (24–43)
FVC % of predicted	53 (40–64)
FEV1/FVC ratio	49.5 (41–69)
DLCO, %	45 (28–57)
6MWT, m	475 (255–540)
Chronic lung infections, n (%)	
*P. aeruginosa*	12 (54.5)
MRSA	6 (27.3)
Aspergillus	2 (9.1)
*C. albicans*	2 (9.1)
*B. cepacia*	1 (4.5)
Medications, n (%)	
Beta-agonists	20 (90.9)
Steroids	18 (81.8)
Oxygen	16 (72.7)
Beta-blockers	3 (13.6)

*n* number, *IQR 25–75* interquartile range, *mPAP* mean pulmonary arterial pressure, *FEV1* forced expiratory volume in 1 s, *FVC* forced vital capacity, *DLCO* diffusing capacity of the lung for carbon monoxide, *6MWT* six minutes walking test, *MRSA* methicillin-resistant *Staphylococcus aureus*

### Acute effects of lung transplant on cardiac autonomic modulation at rest

**Fig. 1:**
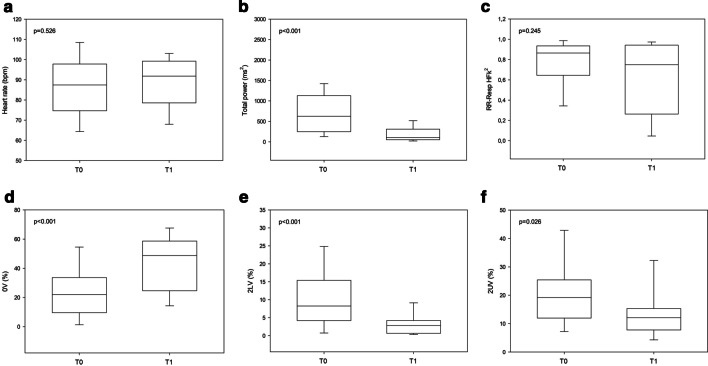
Acute effects of lung transplant on cardiac autonomic modulation from spectral and symbolic analyses in supine position. Total power represents the global heart rate variability (the sum of VLF, LF and HF spectral components); 0 V (%) represents sympathetic contribution; 2UV (%) and 2LV (%) represent autonomic parasympathetic contribution; the squared coherence function at high frequencies between breath rate and heart rate (RR-RESP HFk^2^) represents the cardiorespiratory coupling that ranges from 0 (no correlation) and 1 (highest correlation); bpm: beats per minute; ms: milliseconds; T0: baseline, before lung transplant; T1: 15 days after lung transplant. α < 0.05

Acute effects of lung transplantation were evaluated through a comparison between HRV detections at T0 and T1. Results are reported in Fig. 1.

Spectral analysis showed that at T1, compared to baseline, patients showed significantly lower total power in SUP position [104 (53–310) vs. 625 (247–1130) ms^2^, T1 vs. T0, p < 0.001]. LFnu, HFnu and LF/HF in SUP position did not reach statistical significance (see Additional file 1).

Regarding symbolic analysis, increased 0 V% [43 (19–4) vs. 23 (17–4), p < 0.001] and a decreased 2LV% [3 (1–4) vs. 8 (4–15), p < 0.001] and 2UV% [12 (8–15) vs. 19 (12–25) p < 0.001] were found in T1 compared to T0 in SUP. These results suggest a predominant sympathetic modulation and a lower vagal modulation in post-transplant patients during the acute phase after surgery.

As to cardiorespiratory coupling, comparisons between T0 and T1 did not show statistically significant differences while assessing respiratory frequency, through Resp HF (Hz) [0.34 (0.31–0.38) vs. 0.32 (0.28–0.36), p = 0.444] and cardiorespiratory coupling through RR-Resp HFk^2^ [0.87 (0.65–0.94) vs. 0.75 (0.26–0.94), p = 0.245].

**Fig. 2:**
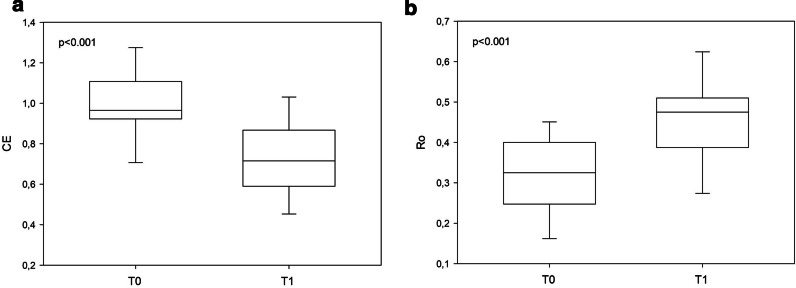
Acute effects of lung transplant on cardiac autonomic complexity from entropy-derived parameters in supine position. Corrected conditional entropy (CE) represents the predictability of R-R intervals (i.e., low predictability = high sympathetic modulation); Index of regularity (Ro) is derived from CE and could range from 0 to 1 (high regularity = low complexity and high sympathetic modulation); bpm: beats per minute; ms: milliseconds; T0: baseline, before lung transplant; T1: 15 days after lung transplant. α < 0.05

As to entropy-derived measures, patients at T1 showed lower CE and higher Ro index than at T0 [0.71 (0.60–0.86) vs. 0.96 (0.93–1.10), p < 0.001; 0.47 (0.39–0.51) vs. 0.32 (0.25–0.40), p < 0.001), revealing higher regularity and lower complexity of HRV in SUP (see Fig. 2).

### Chronic effects of lung transplant on autonomic function

**Fig. 3:**
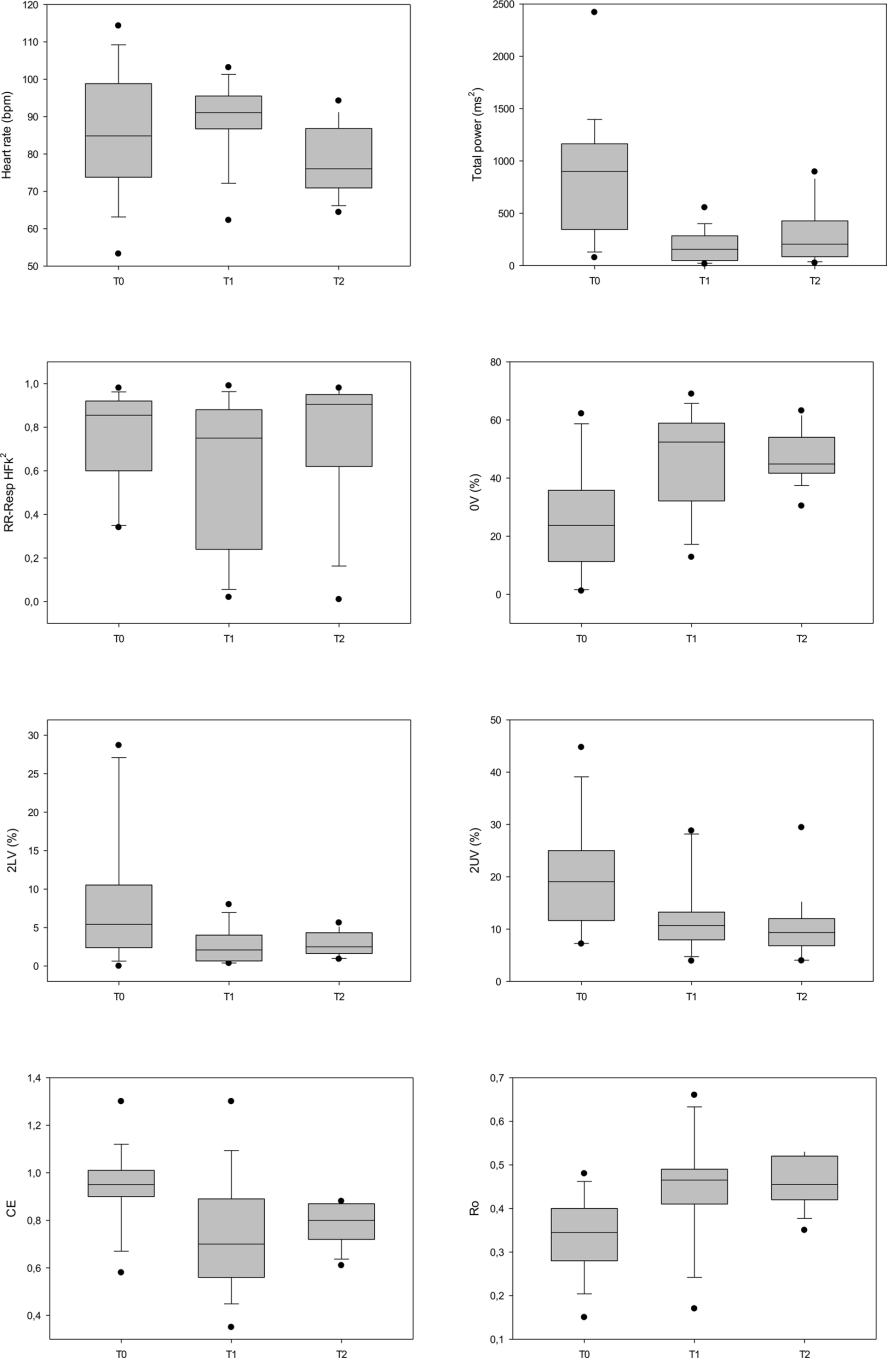
Effects of lung transplant on cardiac autonomic modulation in supine position evaluated at three different time points. Total power represents the global heart rate variability (the sum of VLF, LF and HF spectral components); the squared coherence function at high frequencies between breath rate and heart rate (RR-RESP HFk2) represents the cardiorespiratory coupling that ranges from 0 (no correlation) and 1 (highest correlation); 0 V(%) represents sympathetic contribution; 2UV(%) and 2LV(%) represent autonomic parasympathetic contribution; corrected conditional entropy (CE) represents the predictability of R-R intervals (i.e., low predictability = high sympathetic modulation); Index of regularity (Ro) is derived from CE and could range from 0 to 1 (high regularity = low complexity and high sympathetic modulation); bpm: beats per minute; ms: milliseconds; T0: baseline, before lung transplant; T1: 15 days after lung transplant; T2: 6 months after lung transplant. α < 0.05

Chronic effects of lung transplantation were evaluated through a comparison between HRV detections at T0, T1, and T2 in 14 patients (see Table 2 and Fig. 3). As to spectral parameters, T1 and T2 showed lower total variability compared to T0, and no other statistical difference was found.

**Table 2:** Comparison of autonomic parameters before transplantation (T0), 10–15 days after transplant (T1) and 6 months after surgery (T2). Autonomic parameters were obtained in supine position

	T0 n = 14	T1 n = 14	T2 n = 14	p
Heart rate, median (IQR) bpm	85 (74–99)	91 (87–96)	76 (71–87)	0.099
Spectral analysis, median (IQR)				
Total power, ms^2^	898 (345–1163)	156 (49–284)^a^	204 (85–427)^a^	< 0.001
LFnu	48 (26–69)	42 (14–78)	66 (43–79)	0.508
HFnu	21 (9–66)	19 (8–58)	23 (16–44)	0.94
LF/HF	2.21 (0.37–4.13)	2.64 (0.42–8.44)	2.89 (1.00–5.24)	0.477
RR-RESP HFk^2^	0.86 (0.6–0.92)	0.75 (0.24–0.88)	0.91 (0.62–0.95)	0.408
RESP HF, Hz	0.34 (0.31–0.38)	0.33 (0.28–0.36)	0.29 (0.26–0.33)	0.212
Symbolic analysis, median (IQR)				
0 V%	24 (11–36)	52 (32–59)^a^	45 (42–54)^a^	0.002
2LV%	5 (2–11)	2 (1–4)	3 (2–4)	0.076
2UV%	19 (12–25)	11 (8–13)	9 (7—12)^a^	0.015
Entropy measures, median (IQR)				
CE	0.95 (0.9–1.01)	0.70 (0.56–0.89)^a^	0.8 (0.72–0.87)	0.025
Ro	0.35 (0.28–0.4)	0.47 (0.41–0.49)^a^	0.46 (0.42–0.52)^a^	0.006

*n* number, *LTx* transplant list, *IQR 25–75*, interquartile range, *bpm* beats per minute, *ms*^*2*^ milliseconds^2^, *LF* low frequency, *HF* high frequency, *nu* normalized, *LF/HF* sympatho-vagal balance, *RR* R–R interval, *RESP* respiratory, *K*^*2*^ coherence, *Hz* Hertz, *CE* conditional entropy, *Ro* index of regularity

^a^Differences from T0

Symbolic analysis evidenced higher 0 V% at T1 and T2 than at T0, and lower 2UV% at T2 than at T0, suggesting a stronger sympathetic and a lower vagal modulation in the chronic phase after lung transplantation.

Assessing cardiorespiratory coupling, the breathing rate was coupled with the HF component of HRV with no changes in all three of the time points [HFk^2^ 0.85 (0.60–0.92) vs. 0.75 (0.24–0.88) vs. 0.90 (0.62–0.95), p = 0.408]. Differences in HF component of breath rate between T0, T1 and T2 did not reach statistical significance [Resp HF 0.34 (0.31–0.38) vs. 0.33 (0.28–0.36) vs. 0.29 (0.26–0.33), p = 0.212].

Moreover, entropic measures were performed and showed lower CE at T1 than at T0 and higher Ro index both at T1 and at T2 than at T0, confirming higher regularity and lower complexity of cardiac autonomic modulation also in the chronic phase after surgery.

### Cardiac autonomic response to orthostatic stress

Cardiac autonomic response was evaluated both in the acute phase through T0-T1 comparison and in the chronic phase through T0-T1-T2 comparison. No statistical difference was found as regards all indexes from spectral analysis (Total power; LFnu; HFnu and LF/HF), symbolic analysis (0 V%; 2UV and 2LV%), and entropy-derived measures (CE and Ro) evaluation between the three-time points of recording. Complete data are available in Additional files 2 and 3.

## Discussion

The major findings of the current study are: (1) in the acute phase after BLT, cardiac autonomic modulation is characterized by a reduction of global heart rate variability; (2) the reduction of global heart rate variability is associated with a shift of the autonomic modulation through a sympathetic predominance and a vagal withdrawal and with a decreased complexity of cardiac autonomic modulation; (3) these alterations remain stable across the time after 6 months from the BLT; (4) cardiorespiratory coupling between cardiac and respiratory oscillations are not affected by the BLT procedure in acute and chronic phase; (5) cardiac autonomic responses to orthostatic challenge is not affected by BLT.

To our knowledge, this is the first study that investigated the changes of autonomic modulation before and after BLT. In fact, some studies evaluated the autonomic profile in lung-transplanted patients compared to control groups [7–14]. Most of them presented small sample size and no comparisons between pre and post-surgery and different time points.

Morgan-Hughes et al. [8] evaluated cardiac autonomic profile in the acute phase after lung transplantation (after six to eight weeks) in comparison to the others cited before. The authors conducted some pharmacological and non-pharmacological autonomic tests (hand-grip, Valsalva maneuver, deep breathing and adenosine administration). Cardiac parasympathetic influence during deep breathing was reduced for all and an abnormal Valsalva ratio only in patients underwent surgical vagal cardiac denervation. These findings may have explained by both the deconditioning effect of long-term chronic illness and the lower contribution from pulmonary afferents to respiratory sinus arrhythmia.

Our results reveal that cardiac vagal modulation is acutely reduced after lung transplantation (T1; 15 days after BLT). Even in the acute phase, BLT shifted the cardiac autonomic balance towards a sympathetic predominance and a vagal withdrawal, as demonstrated by the nonlinear HRV method (symbolic dynamics; 0 V% and 2UV%) [16]. The complexity of heart rate dynamics was also changed in BLT acute phase as shown by the increased Ro and decreased CE, both related to high sympathetic modulation [16, 18]. This higher sympathetic modulation to the heart after BLT could be explained, in part, by the loss of vagal afferent fibres from the lung, wasting a reflex vagal buffer on sympathetic drive.

It was shown that double lung transplant recipients could have compatible responses with complete cardiac denervation (i.e., compared to heart–lung transplanted) [6]. Besides, after double lung transplant, patients had an abnormal heart rate and blood pressure responses during autonomic tests (e.g., Valsalva manoeuvre and adenosine administration) [7]. Thus, the cardiac denervation after double lung transplantation could be an underlying consequence of the surgical interruption of sympathetic and parasympathetic pathways [7].

Studies investigating the Hering-Breuer reflex in BLT [9] or heart–lung transplantation [10] showed that in BLT, the abolished Hering-Breuer reflex would be expected because of the interruption of the pulmonary branch of the vagus nerve. It has been observed that intrapulmonary stretch receptors were not reinnervated to allow expression of the classic Hering-Breuer reflex [9]. In contrast to human studies, the reinnervation of intrapulmonary stretch receptors occurred after 1 year following the vagal section in dogs [19, 20]. Thus, evidence of an autonomic reinnervation after lung or heart–lung transplants remain unclear.

HRV is influenced by oscillations from a range of sources, as breathing pattern [3]. The neuronal control of breathing and heart rate are closely linked, and the term cardiorespiratory coupling is often assigned to underlying mechanisms in heart rate fluctuations driven by respiration [3, 21]. Cardiac and respiratory centres are closer functionally, as well as anatomically (i.e., medulla oblongata) and they are critical for survival [22, 23]. The cardiorespiratory control is essential for the regulation of respiratory and cardiac rhythms and the homeostasis of blood gases (i.e., partial pressure differences in oxygen and carbon dioxide), which is related to the breathing depth (tidal volume) and frequency (respiratory cycle) [3, 21]. The cardiorespiratory coupling may be assessed through different mathematical approaches. In an overview, heart rate and respiratory signals must be acquired in a bivariate framework, and the directionality of the interactions must be taken into account to exploit causal relationships in the cardiorespiratory control [17].

In healthy subjects, the cardiorespiratory coupling is preserved, while in pathological conditions this coupling is impaired [24, 25]. In the current study, for the first time, we showed that cardiorespiratory coupling did not change by the BLT procedure in acute and chronic phases, suggesting a maintained coupled control of breathing and circulation.

As regards to HRV indexes, lung transplant presented a lower vagal and sympathetic modulation than the healthy control group [13, 14]. However, these studies have some limitations: (1) the small sample size [12, 14]; (2) the absence of a healthy control group [12]; and (3) the comparison of HRV before and after surgery [12, 14]. From our results, BLT reduced global autonomic modulation to the heart and shifted the autonomic modulation through a sympathetic predominance and a vagal withdrawal in the acute (T1; 15 days after BLT) and the chronic phase (T2; 6 months after BLT) compared to the baseline (T0; before surgery).

The main limits of the present study were the absence of a direct measure of autonomic activity, such as the recording of muscle sympathetic fibres (i.e., Muscle sympathetic nerve activity) and the lack of dynamic autonomic response evaluated by autonomic manoeuvres, except for the active standing. Taken together, these measurements and tests could have added complementary information to cardiovascular autonomic regulation, however, they were not applicable in this population. Also, chemoreflex and Hering-Breuer reflex, such as their relationships with cardiovascular autonomic modulation before and after BLT, should be aim of further investigate. Second, some medications that affect autonomic nervous system were not controlled (e.g. adrenergic agonists/antagonists) and may influenced the final results. Otherwise, all medications used were part of the best medical therapy before and after BLT. Third, we did not perform repetitive measures before the transplant, and we did have some drop-outs due to the presence of exclusion criteria at the timing of recording (i.e. acute events and hospitalization). Finally, different surgical procedures could have an impact on cardiac autonomic modulation. On the other hand, to our knowledge, this is the first study assessing the autonomic profile of a population who underwent BLT before and after transplant in relatively big number of patients and supporting the hypothesis that BLT, although characterized by cardiopulmonary denervation, is not associated with a complete disruption of cardiorespiratory coupling. Studies over a much longer follow up are warranted to evaluate a possible cardiopulmonary reinnervation.

## Conclusion

In conclusion, BLT reduced the global contribution from autonomic nervous system on R-R oscillations as described by global variability and complexity of cardiac autonomic modulation in acute phase. These alterations remain stable after 6 months from surgery. After 6 months from BLT, a sympathetic predominance and a vagal withdrawal could be the marker of partially restored sympathetic oscillations in this population. On the other hand, cardiorespiratory coupling and cardiac autonomic responses to orthostatic challenge seems to be not affected by BLT in acute nor chronic phases.

## Supplementary Information

**Additional file 1: Table S1.** Comparison of autonomic parameters evaluated by spectral analysis before transplantation (T0) and 10-15 days after transplant (T1).

**Additional file 2: Table S2.** Comparison of autonomic dynamic response to orthostatism before transplantation (T0) and 10–15 days after transplant (T1).

**Additional file 3: Table S3.** Comparison of autonomic dynamic response before transplantation (T0), 10–15 days after transplant (T1) and 6 months after surgery (T2).

## Data Availability

The datasets used and analyzed during the current study are available from the corresponding author upon reasonable request.

## Abbreviations

BLT: Bilateral lung transplantation
HRV: Heart rate variability
T0: Before transplantation
T1: Fifteen days after transplantation
T2: Six months after transplantation
Hz: Respiratory frequency
SUP: Supine position
ORT: Orthostatic standing
∆: Delta
LF: The low frequency component
HF: The high frequency component
LFnu: The LF expressed in normalized units
HFnu: The HF expressed in normalized units
LF/HF: The sympatho-vagal balance
0V: Index of three equal symbols
1V: Index of three equal symbols with one variation
2LV: Index of three different symbols with 2 like variations
2UV: Index of three different symbols with 2 like variations
HFk^2^: The squared coherence function at high frequencies
CE: Corrected conditional entropy
Ro: Index of regularity
REM: Rapid eye movement
