# Supplementary material for: Effects of bilateral lung transplantation on cardiac autonomic modulation and cardiorespiratory coupling: a prospective study
Source: Respir Res. 2021 May 21;22:156. doi: 10.1186/s12931-021-01752-6 (PMC8140499; doi:10.1186/s12931-021-01752-6)
Supplement: Supplementary file 1 — Additional file 1: Table S1. Comparison of autonomic parameters evaluated by spectral analysis before transplantation (T0) and 10-15 days after transplant (T1). [file 12931_2021_1752_MOESM1_ESM.docx]

**Supplementary Files 1.** Comparison of autonomic parameters evaluated by spectral analysis before transplantation (T0) and 10-15 days after transplant (T1).

|  | T0  n = 22 | T1  n = 22 | p |
| --- | --- | --- | --- |
| Heart rate, median (IQR) bpm | 86 (± 16) | 89 (± 13) | 0.526 |
| Spectral analysis, median (IQR) |  |  |  |
| Total power, ms^2^ | 625 (247 - 1130) | 104 (53 - 310) | <0.001 |
| LFnu | 54 (25 - 77) | 40 (6 - 74) | 0.342 |
| HFnu | 21 (9 - 57) | 20 (10 - 57) | 0.897 |
| LF/HF | 2.24 (0.43 - 6.34) | 2.64 (0.32 - 7.80) | 0.743 |

n, number; LTx, transplant list; IQR 25-75, interquartile range; SUP, supine; bpm, beats per minute; ms^2^, milliseconds^2^; LF, low frequency; HF, high frequency; nu, normalized; LF/HF.
