# Supplementary material for: Effects of bilateral lung transplantation on cardiac autonomic modulation and cardiorespiratory coupling: a prospective study
Source: Respir Res. 2021 May 21;22:156. doi: 10.1186/s12931-021-01752-6 (PMC8140499; doi:10.1186/s12931-021-01752-6)
Supplement: Supplementary file 2 — Additional file 2: Table S2. Comparison of autonomic dynamic response to orthostatism before transplantation (T0) and 10–15 days after transplant (T1). [file 12931_2021_1752_MOESM2_ESM.docx]

Supplementary Files 2. Comparison of autonomic parameters before transplantation (T0) and 10-15 days after transplant (T1). Autonomic dynamic response to orthostatism is displayed as percentile (∆ORT = (HRV in SUP position − HRV in ORT position)/HRV in SUP position).

|  | T0  n = 22 | T1  n = 22 | p |
| --- | --- | --- | --- |
| Heart rate, median ∆ORT % | 12 (9 - 15) | 11 (7 - 12) | 0.343 |
| Spectral analysis, median ∆ORT % |  |  |  |
| Total power, ms^2^ | -18 (-51 - 9) | -11 (-53 - 76) | 0.935 |
| LFnu | 29 (5 - 193) | 71 (-5 - 342) | 0.490 |
| HFnu | -19 (-65 - 43) | -58 (-82 - -16) | 0.118 |
| LF/HF | 103 (-27 - 650) | 745 (17 - 3004) | 0.132 |
| RR-RESP HFk^2^ | -4 (-18 - 7) | -8 (-63 - 5) | 0.323 |
| RESP HF, Hz | 1 (-11 - 15) | 3 (-10 - 17) | 0.987 |
| Symbolic analysis, median ∆ORT % |  |  |  |
| 0V% | 56 (9 - 181) | 5 (-24 - 144) | 0.083 |
| 2LV% | -17 (-51 - 64) | 66 (-85 - 131) | 0.360 |
| 2UV% | -23 (-55 - 10) | -23 (-58 - 27) | 0.685 |
| Entropy measures, median ∆ORT % |  |  |  |
| CE | -6 (-14 - 3) | 7 (-26 - 47) | 0.203 |
| Ro | 21 (-4 - 57) | 14 (-14 - 30) | 0.297 |

n, number; Δ, delta; ORT, orthostatism; SUP, supine; ms^2^, milliseconds^2^; LF, low frequency; HF, high frequency; nu, normalized; LF/HF, sympathovagal balance; RR, R-R interval; RESP, respiratory; K^2^, coherence; Hz, Hertz; CE, conditional entropy; Ro, index of regularity.
