# Supplementary material for: Effects of bilateral lung transplantation on cardiac autonomic modulation and cardiorespiratory coupling: a prospective study
Source: Respir Res. 2021 May 21;22:156. doi: 10.1186/s12931-021-01752-6 (PMC8140499; doi:10.1186/s12931-021-01752-6)
Supplement: Supplementary file 3 — Additional file 3: Table S3. Comparison of autonomic dynamic response before transplantation (T0), 10–15 days after transplant (T1) and 6 months after surgery (T2). [file 12931_2021_1752_MOESM3_ESM.docx]

Supplementary Files 3. Comparison of autonomic parameters before transplantation (T0), 10-15 days after transplant (T1) and 6 months after surgery (T2). Autonomic dynamic response to orthostatism is displayed as percentile (∆ORT = (HRV in SUP position − HRV in ORT position)/HRV in SUP position).

|  | T0  n = 14 | T1  n = 14 | T2  n = 14 | p |
| --- | --- | --- | --- | --- |
| Heart rate, median ∆ORT % | 11 (± 4) | 11 (± 5) | 14 (± 9) | 0.256 |
| Spectral analysis, median ∆ORT % |  |  |  |  |
| Total power, ms^2^ | 27 (± 112) | 50 (± 152) | -18 (± 62) | 0.279 |
| LFnu | 145 (± 208) | 93 (± 158) | 44 (± 103) | 0.300 |
| HFnu | -28 (± 44) | -40 (± 83) | -16 (± 84) | 0.694 |
| LF/HF | 925 (± 2274) | 1616 (± 2837) | 893 (± 1240) | 0.665 |
| RR-RESP HFk^2^ | -12 (± 34) | 18 (± 130) | -1 (± 36) | 0.532 |
| RESP HF, Hz | -7 (± 31) | -3 (± 38) | -1 (± 35) | 0.880 |
| Symbolic analysis, median ∆ORT % |  |  |  |  |
| 0V% | 100 (± 256) | 32 (± 85) | -5 (± 43) | 0.260 |
| 2LV% | 49 (± 116) | 61 (± 282) | -12 (± 80) | 0.436 |
| 2UV% | -12 (± 50) | 25 (± 116) | 23 (± 98) | 0.708 |
| Entropy measures, median ∆ORT % |  |  |  |  |
| CE | -2 (± 11) | 5 (± 36) | -4 (± 19) | 0.639 |
| Ro | 15 (± 29) | 27 (± 56) | 10 (± 17) | 0.626 |

n, number; Δ, delta; ORT, orthostatism; SUP, supine; ms^2^, milliseconds^2^; LF, low frequency; HF, high frequency; nu, normalized; LF/HF, sympathovagal balance; RR, R-R interval; RESP, respiratory; K^2^, coherence; Hz, Hertz; CE, conditional entropy; Ro, index of regularity.
